# Modulation by Phosphonium Ions of the Activity of Mitotropic Agents Based on the Chemiluminescence of Luminols

**DOI:** 10.3390/molecules27041245

**Published:** 2022-02-12

**Authors:** Gemma M. Rodríguez-Muñiz, Theodoros Mikroulis, Anna Pantelia, Georgios Rotas, Maria-Consuelo Cuquerella, Georgios C. Vougioukalakis, Miguel A. Miranda

**Affiliations:** 1 Instituto de Tecnología Química UPV-CSIC, Universitat Politècnica de València, Camino de Vera s/n, 46022 València, Spain; gemrodmu@itq.upv.es (G.M.R.-M.); xecual@gmail.com (M.-C.C.); 2 Laboratory of Organic Chemistry, Department of Chemistry, National and Kapodistrian University of Athens, Panepistimiopolis, 15771 Athens, Greece; theo.mik0@gmail.com (T.M.); annapantelia@gmail.com (A.P.); rotasgiorgos@hotmail.com (G.R.)

**Keywords:** luminols, chemiluminescence, 3-aminophthalates, fluorescence, electron transfer

## Abstract

Mitochondria-targeting drugs and diagnostics are used in the monitoring and treatment of mitochondrial pathologies. In this respect, a great number of functional compounds have been made mitotropic by covalently attaching the active moiety onto a triphenylphosphonium (**TPP**) cation. Among these compounds, a number of molecular detectors for reactive oxygen species (ROS) are based on fluorescent and chemiluminescent probes. In this regard, luminol (probably the most widely known chemiluminescent molecule) has been employed for a number of biological applications, including ROS detection. Its oxidation under specific conditions triggers a cascade of reactions, ultimately leading to the excited 3-aminophthalate (3AP *), which emits light upon deactivation. Hence, the photophysical interaction between the light-emitting species 3AP * and **TPP** cations needs to be evaluated, as it can add valuable information on the design of novel emission-based mitotropic systems. We herein investigate the quenching effect of ethyltriphenylphosphonium cation onto substituted 3-aminophthalates. These were prepared in situ upon hydrolysis of the corresponding anhydrides, which were synthesized from 3-aminophthalimides. Steady-state fluorescence and time-resolved experiments were employed for the evaluation of a possible electron transfer quenching by phosphonium ions. Our experimental results confirmed such quenching, suggesting it is mainly dynamic in nature. A minor contribution of static quenching that was also detected is attributed to complex formation in the ground state. Accordingly, the chemiluminescence of luminol was indeed strongly reduced in the presence of phosphonium ions. Our results have to be taken into account during the design of new chemiluminescent mitotropic drugs or diagnostic agents of the luminol family**.**

## 1. Introduction

Mitochondria are acknowledged as the subcellular organelles directly affecting both life, as cell’s energy powerhouses, and death, as apoptosis regulators [1,2,3]. Up to 90% of cells’ energy unit ATP [4] and cytotoxic ROS [5] are generated in mitochondria. As a result, mitochondrial malfunction, causing ROS overproduction, is connected to pathogenesis, such as neurodegenerative diseases, cancer, and diabetes [6,7,8].

Rendering drugs and diagnostics mitotropic (or mitochondriotropic), that is, achieving organelle-specific accumulation, is a way for monitoring and treating mitochondrial pathologies. The large potential difference between the intermembrane space and the mitochondrial matrix (negative in the matrix) makes delocalized lipophilic cations ideal mitotropic carriers [9]. Among these, triphenylphosphonium cations (TPPCs) stand out, both due to their exceptional mitotropic activity and ease of synthesis. In this respect, a great number of active compounds, either drugs or diagnostics, have been made mitotropic by covalently attaching the active compound onto a TPPC moiety [10,11,12,13].

**Figure 1:**
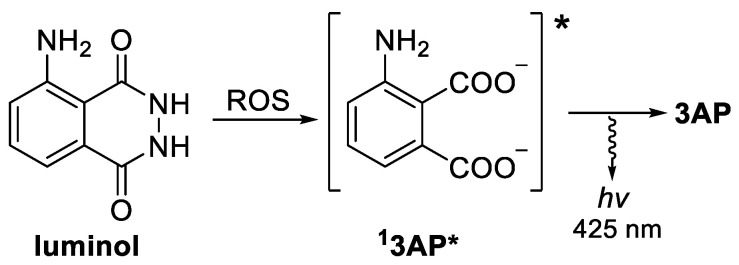
ROS triggered luminol CL reaction, producing **3AP** and light.

The importance of ROS imbalance in oxidative stress-caused pathology has triggered research in sensors for intracellular ROS levels detection. As a result, a number of ROS molecular detectors based on fluorescent and chemiluminescent (CL) probes have been prepared and evaluated [14,15,16,17]. Although both are based on light emission, CL probes exhibit advantages, such as a high signal-to-noise ratio and no need for external irradiation [18]. In this regard, luminol, probably the best known CL molecule [19], has also been widely studied in vivo, both as ROS detector, and excitation light source, due to its strong CL, stability, and ease of synthesis [20,21,22,23]. Its reaction with ROS (especially superoxide anion) in the presence of a catalyst triggers a chain reaction, ultimately leading to excited 3-aminophthalate (3AP *, Figure 1), which emits light upon deactivation.

In the framework of our research towards mitotropic, highly efficient, luminol-based, CL ROS detectors, consisting of covalently attached TPPCs onto luminol via a linker [24], the photophysical interaction between the light-emitting species 3AP * and TPPC needs to be evaluated as it can add valuable information on the design of novel emission-based mitotropic systems. To the best of our knowledge, the photophysical evaluation of TPPCs as aminophthalate quenchers is presented here for the first time.

## 2. Results and Discussion

**Figure 2:**
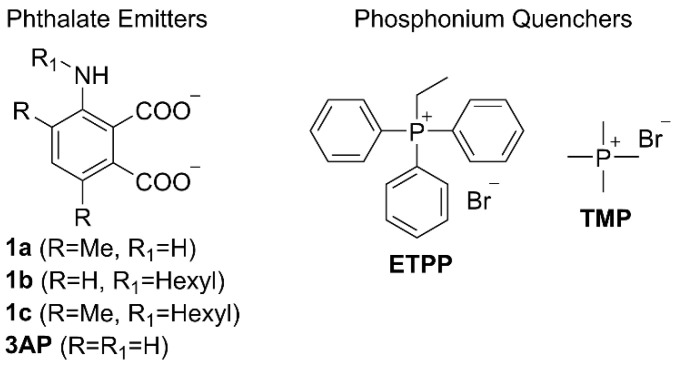
Structures of the phthalate emitters and phosphonium quenchers studied.

**Figure 3:**
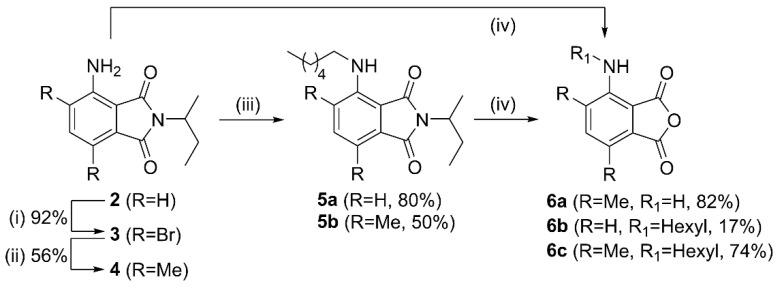
Synthesis of phthalic acid anhydride derivatives **6a**–**c**. Reagents and conditions: (**i**) Br_2_, HOAc, NaOAc, r.t., 18 h, (**ii**) trimethylboroxine, Pd(PPh_3_)_4_, K_2_CO_3_, H_2_O, 1,4-dioxane 105^o^C, 18 h, (**iii**) 1-iodohexane, NMP, 110 °C, 2d, (**iv**) 15N aq. KOH, EtOH, reflux, 3 d.

We have chosen to investigate the quenching effect of the ethyltriphenylphosphonium (**ETPP**) cation onto phthalates **1a–c** and **3AP** (Figure 2). **ETPP** is a representative mitotropic, delocalized, lipophilic cation, whereas tetramethylphosphonium (**TMP**) is used here as a reference, non-delocalized cation. Phthalate structures **1a**–**c** were chosen in order to evaluate the results pertaining to **3AP** core substitution, which have been shown to greatly affect the corresponding luminol CL efficiency [25,26,27]. The preparation of phthalates **1a**–**c** was accomplished as follows: 3-aminophthalimide **2** was brominated using excess bromine in acetic acid towards phthalimide **3** (Figure 3). This was then functionalized via a Suzuki–Miyaura coupling with trimethylboroxine to give the respective dimethyl phthalimide **4**. *N*-Alkylation of **2** and **4** with 1-iodohexane afforded the *N*-hexyl derivatives **5a**,**b**. Hydrolysis of the phthalimides **4** and **5a**,**b** turned out to be challenging, requiring prolonged reaction times under strongly alkaline conditions. Interestingly, acidic work-up of the crude mixture resulted in the formation of the respective anhydrides **6a**–**c**, instead of the corresponding phthalic acids, indicating an in situ intramolecular condensation. These anhydrides were found to hydrolyze in basic aqueous media towards the corresponding phthalates **1a**–**c**, so they were used as their in situ precursors (see Figure S1 in the Supplementary Materials).

**Figure 4:**
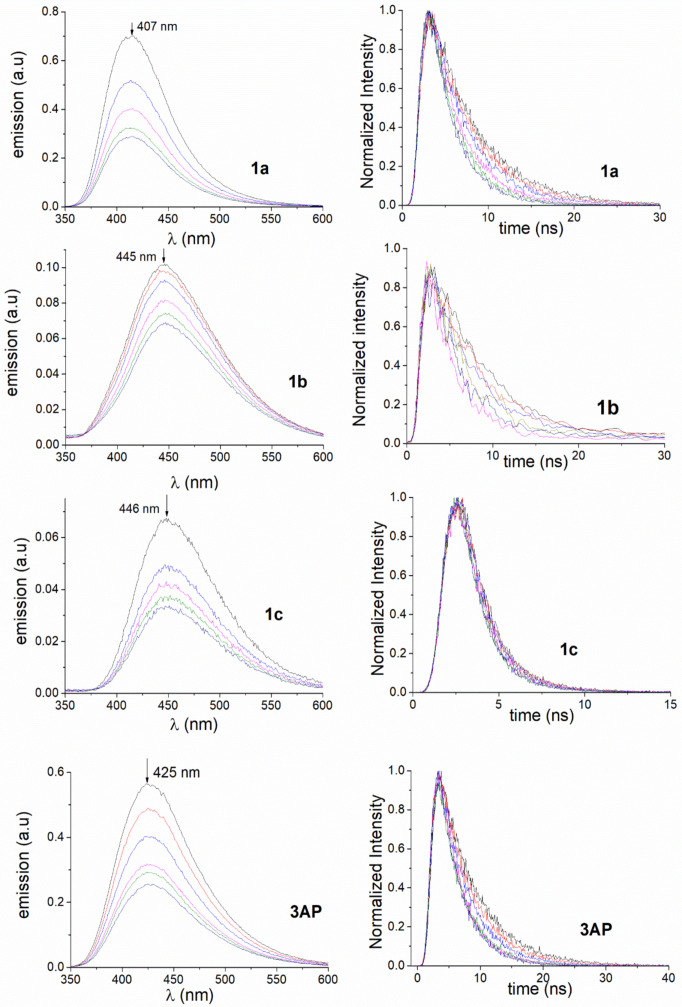
Emission spectra (**left**) and fluorescence decays (**right**) for compounds **1a**–**c** and **3AP** obtained in PBS (0.1 mM) in the presence of increasing amounts of **ETPP** (0–42.1 mM; colour codes: 0 mM black line, 4.6 mM red line, 11.4 mM blue line, 22.2 mM pink line, 32.4 mM green line, 42.1 mM navy blue line).

Steady-state fluorescence and time-resolved experiments were performed for compounds **1a**–**c** and **3AP** in phosphate-buffered saline (PBS) at pH 8 to evaluate the possibility of an electron transfer quenching process from the phthalates to the phosphonium cation, using **ETPP** as the source of phosphonium. The obtained results are shown in Figure 4, Figure S2 in the Supplementary Material, and Table 1. The **TMP** salt was used as a reference to analyze the effect of the triphenyl-substituent in the quenching process (see Table 1 and Figure S3 in the Supplementary Materials).

Τhe marked weakening of phthalate fluorescence upon 3-amino alkylation (**1b,c** vs. **3AP** and **1a**) is an interesting observation. Regarding phosphonium-induced quenching, the values shown in the table indicate that the quenching is mainly dynamic. The contribution of static quenching (difference between the k_q_ and k′_q_ series) can, in principle, be attributed to complex formation in the ground state. This effect is less pronounced when there are methyl groups on the aromatic ring and/or an alkyl chain on the amino group, probably due to steric hindrance. The possibility of an apparent static quenching associated with a filter effect can be safely ruled out based on the lack of absorbance variation at the excitation wavelength (300 nm) upon addition of the phosphonium salt (see Figure S1 in the Supplementary Materials). The fact that electron-donating alkyl substituents, both on the aromatic ring and the amino group, increase the dynamic quenching rate constant is consistent with an electron transfer mechanism. The **TMP** reference phosphonium salt also quenched the fluorescence of **3AP**, but the rate constant was ca. one order of magnitude lower.

**Figure 5:**
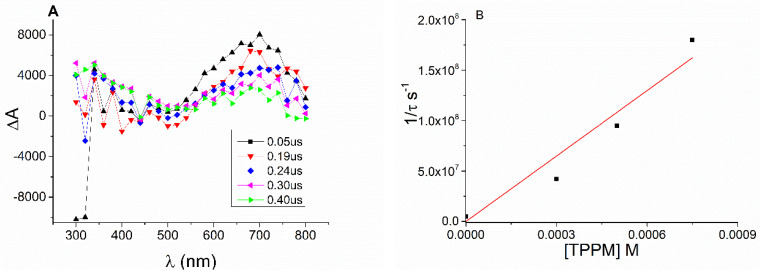
(**A**) Transient absorption spectra of a N_2_-bubbled PBS solution of **3AP** at pH 8 at different times after the 355 nm laser pulse. (**B**) Stern–Volmer plot for quenching of the solvated electron by **ETPP** (from 0 to 0.8 mM) under N_2_.

The generation of short-lived intermediates from **3AP** in the presence of **ETPP** was investigated by laser flash photolysis (LFP). The transient absorption spectra were monitored upon laser excitation (λ = 355 nm) in deaerated PBS at pH 8. They revealed the formation of a broad shapeless band with a maximum in the 700 nm region (Figure 5A). This species was ascribed to the solvated electron, based on its disappearance upon bubbling with N_2_O (Figure 5A inset). This observation is proof for photoionization, which was found to be monophotonic by plotting the signal intensity immediately after the laser pulse versus the pulse energy (see Figure S4 in the Supplementary Materials). As expected, the solvated electron was quenched by oxygen with a k_q_ = 5.8 × 10^9^ M^−1^s^−1^ (see Figure S5 in the Supplementary Materials).

To evaluate the reactivity of the solvated electron with **ETPP,** the quenching rate constant was determined. Hence, the decay was monitored at 700 nm upon the addition of increasing amounts of **ETPP** (Figure 5B inset). The corresponding rate constant value was obtained from the slope of the linear plot by representing reciprocal lifetime versus **ETPP** concentrations (Figure 5B). The quenching rate constant obtained for the reaction of the solvated electron with **ETPP** was k_q_ = 2.2 × 10^8^ M^−1^s^−1^.

The above results demonstrate that photoionization of **3AP** produces solvated electrons which react readily with **ETPP**, hence leading to a reduction of the latter. In order to evaluate the feasibility of a direct electron transfer process in the excited state, we made an estimation of the ΔG associated to the process by means of the Rehm–Weller equation (Equation (3)).

^1^**3AP** * → **3AP**^+^ + 1e^−^ (1)

**ETPP**^+^ + 1e^−^ → **ETPP** (2)

ΔGet(kcal mol^−1^) = −23.06 × [E_red_(**ETPP**^+^/**ETPP**) − E_red_(**3AP**^+^/**3AP**)] − E_S_(**3AP**) (3)

The singlet excited state energy (E_S_) was determined ca. 72.4 kcal mol^−1^ from the intersection between the normalized emission and excitation spectra. (see Figure S6 in the Supplementary Materials).

The reduction potentials were experimentally determined by cyclic voltammetry (see Figure S7 in Supplementary Materials), namely: E_red_(**ETPP**^+^/**ETPP**) = −0.86 V and E_red_(**3AP**^+^/**3AP**)] = 0.68 V.

Using the obtained values for E_S_ and E_red,_ the resulting ΔG for the electron transfer process from the singlet excited state was −37.0 kcal mol^−1^ indicating that the process is indeed thermodynamically favorable.

**Figure 6:**
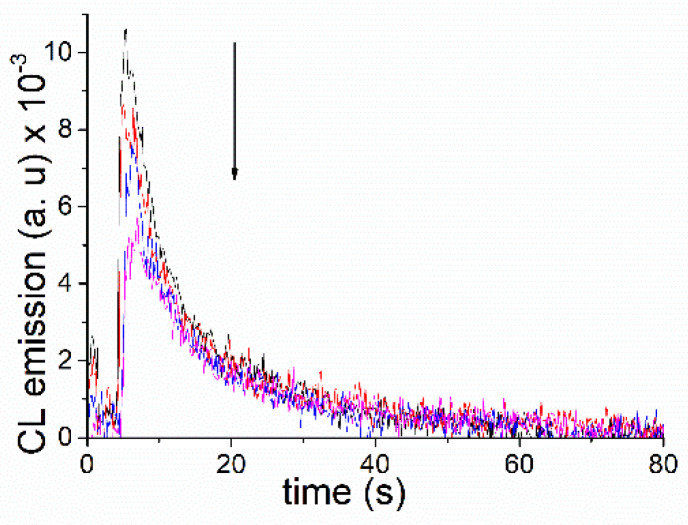
Reduction of luminol’s chemiluminescence (7.5 μM) in the presence of increasing amounts of ETPP (0–7.8 mM) in aqueous solutions at pH 10.

To assess the impact of phosphonium quenching on the CL of the commercially available luminol, this was dissolved in aqueous solutions at pH 10, giving a final concentration of 7.5 μM. Two milliliters were placed in a quartz cuvette, and the CL was manually triggered by the subsequent addition of H_2_O_2_ and K_3_[Fe(CN)_6_] while vigorously stirring (Figure 6; black trace). Monitoring of the process was performed using a fluorometer running in the time-based mode (own lamp switched off, 425 nm as monitoring wavelength). Parallel experiments were carried out where the CL of luminol was measured in the presence of increasing amounts of **ETPP** (0–7.8 mM, concentration in the cuvette, colored traces). As a matter of fact, a consistent reduction of luminol´s CL in the presence of the phosphonium cation was observed. This is in agreement with the results discussed above on fluorescence quenching of the phthalate emitters by **ETPP**.

In summary, phosphonium cations interact with the singlet excited state of phthalates, leading to a partial quenching of their fluorescence. The mechanism is attributed to an excited state electron transfer based on the substituent effects and on the favorable thermodynamics of the process, according to the Rehm-Weller equation. The observed quenching has an impact on the chemiluminescence of the precursor luminols, reducing their efficiency.

The question of whether this intermolecular effect at high phosphonium concentrations can be extrapolated to a possible intramolecular effect in diluted phosphonium-functionalized luminols remains to be investigated. It can be envisaged that, when linked together with the cation, luminol might be more efficiently quenched. However, obvious ways to circumvent (or at least mitigate) this possible drawback would be to play with the nature of the phosphonium cation (e.g., alkyl-substituted instead of aryl) or with the length of the linking bridge between the luminol core and the phosphonium moiety (the longer, the better).

In addition, these results emphasize the importance of finding improved luminol derivatives with higher chemiluminescence quantum yields so that the remaining emission after a possible partial quenching is still sufficient for the intended purpose.

## 3. Materials and Methods

### 3.1. General Remarks

All chemicals were obtained from commercial sources and were used without further purification. Solvents were dried according to published procedures [28]. The course of the reactions was monitored with thin-layer chromatography (TLC), using aluminum sheets (0.2 mm) coated with silica gel 60 with fluorescence indicator (silica gel 60 F254). Purification of the products was carried out by flash column chromatography, using silica gel 60 (230−400 mesh). Nuclear magnetic resonance (NMR) spectra were obtained with a Bruker Avance 400 MHz (Bruker BioSpin MRI GmbH, Ettlingen, Germany) or a Varian Mercury 200 MHz spectrometer (Varian Inc., Yarnton, UK). Chemical shifts are reported in ppm. High-resolution mass spectral (HRMS) spectra were recorded in a QTOF maXis impact (Bruker) spectrometer under electron spray ionization conditions (the ^1^H and ^13^C NMR data and spectra, as well as HRMS data, are reported in Supplementary Materials, Figure S8).

### 3.2. Photophysical Studies

Steady-state and time-resolved fluorescence measurements were performed with a FLS1000 spectrofluorometer (Edinburgh Instruments, Livingston, UK), equipped with an N-DMM double-emission monochromator, an N-G11 PMT-980 detector, and equipped with a pulsed LED (λ_exc_ 300 nm) as an excitation source. The kinetic traces were fitted by one monoexponential decay function, using a deconvolution procedure to separate them from the lamp pulse profile. All experiments were performed in a quartz cuvette of 1 cm of optical path. These experiments were performed in a PBS solution at pH 8. The phthalate solutions were prepared with a concentration of 10^−4^ M, while a stock solution of **ETPP** (0.4 M) was prepared, so it was only necessary to add microliter volumes (250 μL) to the sample cell to obtain the appropriate concentration of the quencher. The rate constants (k_q_ and k′_q_) for the reaction were obtained from the Stern–Volmer plots following Equations (4) and (5), respectively:

I_0_/I = 1 + k_q_ × τ_0_ × [**ETPP**] (4)

τ_0_/τ = 1 + k′_q_ × τ_0_ × [**ETPP**] (5)

where I_0_ and I are the intensities at the emission maxima in the absence of, and after addition of, a quencher concentration [**ETPP**], τ_0_ is the fluorescence lifetime of the phthalate in the absence of **ETPP** and τ is the lifetime after addition of a quencher concentration [**ETPP**].

Monitoring the CL of luminol at pH 10, in the absence and presence of **ETPP** was performed using the same spectrofluorometer with its own lamp switched off. The set was run in the time-based mode with the detection dialed at 425 nm. Each experiment was performed at least 10 times. For triggering the chemiluminescence, luminols were dissolved in aqueous basic solutions giving a final concentration of 7.5 μM. Then, 2 mL of luminol or luminol plus **ETPP** were introduced in a quartz cuvette and the CL was manually triggered by the addition of 2.5 μL of H_2_O_2_ (50% *w*/*w*) and 8 μL of K_3_[Fe(CN)_6_] 75 mM while vigorously stirring.

### 3.3. Laser Flash Photolysis Studies

For the detection of transient species, time-resolved kinetic analyses were performed using a laser flash photolysis (LFP) system equipped with a Nd: YAG SL404G-10 Spectron Laser (Lotis Tii, Minsk, Belarus) at the excitation wavelength of 355 nm. The single pulses were of ca. 10 ns duration, and the energy was lower than 30 mJ per pulse. The detecting light source was a pulsed Lo255 Oriel Xenon lamp. In addition to the pulsed laser, the LFP included the pulsed Lo255 Oriel Xe lamp (Newport, Irvine, CA, USA), a 77,200 Oriel monochromator, a photomultiplier (Oriel, model 70705PMT) system, and a TDS-640A Tektronix oscilloscope (Betashire, UK). A customized Luzchem Research LFP-111 system was employed to collect and transfer the output signal from the oscilloscope to a personal computer to process the data. A quartz cell of 1 cm optical path length was employed for all kinetic measurements. These experiments were performed in a PBS solution at pH 8, under N_2_ air (0.0019 M O_2_ concentration in solution) and oxygen (0.0091 M O_2_ effective concentration in solution). The phthalate solutions were prepared with a concentration of 0.0016 M (absorbance at λ_exc_ = 355 nm was 0.25), while a stock solution of **ETPP** (0.04 M) was prepared.

### 3.4. Electrochemical Studies

Cyclic voltammetry measurements were performed with a VersaSTAT 3 potentiostat (Princeton Applied Research, Algete, Madrid, Spain) and using a three-electrode standard configuration with a carbon sheet as working electrode, a platinum wire as a counter electrode, and Ag/AgCl in saturated KCl as the reference electrode. Measurements were carried out on DMF or PBS at pH 8 solutions with 0.1 M Bu_4_NI or LiClO_4_ as the electrolyte of ETPP or **3AP** (1 mM) respectively at a scan rate of 0.05 V·s^−1^. All the solutions were previously purged with N_2_ for at least 15 min before the measurements.

### 3.5. Synthesis Procedures

4-Amino-2-(*sec*-butyl)isoindoline-1,3-dione **2** was synthesized as previously published [24]. All derivatives were characterized by ^1^H and ^13^C-NMR and ESI-HRMS.

#### 3.5.1. 4-Amino-5,7-dibromo-2-(*sec*-butyl)isoindoline-1,3-dione (**3**)

A round-bottom flask was charged with aminophthalimide **2** (1 g, 4.6 mmol) and sodium acetate (762 mg, 9.3 mmol) in acetic acid (11 mL), and the resulting solution was stirred at r.t. for 30 min. Then, a solution of bromine (0.5 mL, 9.4 mmol) in acetic acid (4 mL) was added dropwise, and the reaction mixture was stirred at r.t. for 18 h. After that, the mixture was poured into ice-cold water (100 mL), forming a yellow precipitate. This was collected by filtration, washed with water and dried under reduced pressure, yielding compound **3** as a yellow powder (1.6 g, 92%). **^1^H NMR** (200 MHz, CDCl_3_) δ 7.79 (s, 1H, ArH), 5.77 (bs, 2H, NH_2_), 4.34–4.05 (m, 1H, NCH), 2.19–1.89 (m, 1H, CHCH_2_CH_3_), 1.89–1.63 (m, 2H, CHCH_2_CH_3_), 1.44 (d, *J* = 6.9 Hz, 4H, CH_3_CH), 0.87 (t, *J* = 7.4 Hz, 4H, CH_2_CH_3_). **^13^C NMR** (50 MHz, CDCl_3_) δ 168.38, 165.97, 142.23, 141.02, 128.38, 115.62, 113.53, 103.86, 49.17, 26.71, 18.34, 11.32. Molecular ion could not be detected on ESI-MS or ESI-HRMS.

#### 3.5.2. 4-Amino-5,7-dimethyl-2-(*sec*-butyl)isoindoline-1,3-dione (**4**)

A round-bottom flask under an inert atmosphere was charged with brominated phthalimide **3** (1.5 g, 4 mmol), trimethylboroxine (2.5 M in THF, 2.5 mL, 8.8 mmol), and potassium carbonate (3.3 g, 24.2 mmol) in a mixture of water (22 mL) and 1,4-dioxane (22 mL). The solution was purged with argon for approximately 30 min. Tetrakis(triphenylphosphine)palladium(0) (92 mg, 0.08 mmol) was then added and the mixture was purged once again for 15 min. Subsequently, the reaction mixture was stirred under an inert atmosphere at 105 °C for 18 h. After cooling to ambient temperature, the solvent was partially evaporated and water (80 mL) was added to the flask. The aqueous phase was extracted with EtOAc (150 mL). The organic phase was then washed with 1N HCl (50 mL) and brine (50 mL), dried over anhydrous MgSO_4_, filtered and concentrated under reduced pressure. The residue was subjected to column chromatography (P.E./EtOAc 9:1 to 7:3) and, after evaporation of the solvent, **4** was acquired as a yellow solid (552 mg, 56%). **^1^H NMR** (200 MHz, CDCl_3_) δ 6.98 (s, 1H, ArH), 5.17 (bs, 2H, NH_2_), 4.13 (q, *J* = 6.7 Hz, 1H, NC*H*), 2.44 (s, 3H, CH_3_), 2.15 (s, 3H, CH_3_), 1.99 (m, 1H, NCHC*H*_2_), 1.70 (m, 1H, NCHC*H*_2_), 1.39 (d, *J* = 6.9 Hz, 3H, C*H*_3_CH), 0.82 (t, *J* = 7.4 Hz, 3H, CH_2_C*H_3_*). **^13^C NMR** (50 MHz, CDCl_3_) δ 170.86, 169.46, 142.26, 138.04, 129.54, 126.37, 111.02, 48.32, 29.72, 26.96, 18.57, 16.80, 16.42, 11.38. **ESI-HRMS** *m*/*z* for C_14_H_19_N_2_O_2_ [M+H]^+^ calcd. 247.1447, found 247.1429.

#### 3.5.3. General Procedure for the *N*-alkylation of 4-aminophthalimides (**5a**–**5b**)

Aminophthalimide **2** or **4** (2.72 mmol) was dissolved in *N*-methyl-pyrrolidone (0.6 mL). 1-Iodohexane (0.5 mL, 3.3 mmol) was added and the reaction mixture was stirred at 110 °C for two days. After cooling down to ambient temperature, the mixture was quenched with water and was extracted with EtOAc. The organic phase was separated, dried over anhydrous Na_2_SO_4_, filtered and concentrated in vacuum. The residue was subjected to column chromatography (P.E./EtOAc 9:1) and the product (eluted first) was acquired after evaporation of the solvent.

2-(*sec*-Butyl)-4-(hexylamino)isoindoline-1,3-dione (**5a**)**:** From aminophthalimide **2** (594 mg). Yellow oil (659 mg, 80%). **^1^H NMR** (200 MHz, CDCl_3_) δ 7.60 (t, *J* = 7.3 Hz, 1H, H-6), 7.15 (d, *J* = 7.2 Hz, 1H, H-5), 6.94 (d, *J* = 8.8 Hz, 2H, H-7), 6.12 (bs, 1H, NH), 3.29 (t, *J* = 5.7 Hz, 2H, NHC*H*_2_), 1.81–1.57 (m, 2H, NHCH_2_C*H*_2_), 1.55–1.17 (m, 8H, CH_2_), 0.90 (t, *J* = 6.1 Hz, 3H, CH_3_). **^13^C NMR** (50, CDCl_3_) δ 171.22, 168.45, 146.53, 135.39, 132.87, 115.91, 110.47, 110.36, 48.51, 42.68, 31.57, 29.31, 26.98, 26.71, 22.60, 18.54, 14.05, 11.34. **ESI-HRMS** *m*/*z* for C_18_ H_27_N_2_O_2_ [M+H]^+^ calcd. 303.2073, found 303.2078.

2-(*sec*-Butyl)-4-(hexylamino-5,7-dimehtylisoindoline-1,3-dione (**5b**)**:** From aminophthalimide 4 (670 mg). Yellow oil (450 mg, 50%). **^1^H NMR** (200 MHz, CDCl_3_) δ 7.03 (s, 1H, ArH), 6.09 (bs, 1H, NH), 4.28–4.03 (m, 1H, NC*H*), 3.29 (t, *J* = 6.7 Hz, 2H, NHC*H*_2_), 2.48 (s, 3H, CH_3_), 2.36 (s, 3H, CH_3_), 2.13–1.90 (m, 1H, CHC*H*_2_), 1.86–1.68 (m, 1H, CHC*H*_2_), 1.68–1.49 (m, 2H, NHCH_2_C*H*_2_), 1.42 (d, *J =* 6.9 Hz, 3H, C*H*_3_CH), 13.5–1.21 (m, 6H, -CH_2_-), 0.96–0.77 (m, 6H, hexyl-CH_3_ and -CH_2_C*H*_3_). **^13^C NMR** (50 MHz, CDCl_3_) δ 171.21, 169.01, 146.34, 140.25, 133.38, 127.34, 125.66, 114.93, 48.47, 47.14, 31.66, 31.09, 27.03, 26.59, 22.68, 20.59, 18.61, 16.55, 14.96, 12.04. **ESI-HRMS** *m*/*z* for C_20_ H_31_N_2_O_2_ [M+H]^+^ calcd. 330.2307, found 303.2302.

#### 3.5.4. General Procedure for the Synthesis of 3-Aminophthalic Anhydrides (**6a**–**6c**)

To a solution of aminophthalimide (0.3 mmol) in ethanol (3mL) was added an aqueous solution of potassium hydroxide (15 N, 3 mL) and the resulting solution was heated at reflux for 3 days. After cooling to ambient temperature, ethanol was evaporated, water (10 mL) was added to the flask and the solution was washed with DCM (3 × 15 mL). The aqueous phase was collected and acidified with an aqueous solution of 1N HCl until pH 2. Upon acidification, the aqueous solution slowly turned from colourless to fluorescent green, indicative of the condensation of the phthalic acid to the corresponding anhydride. The solution was then extracted with EtOAc (3 × 20 mL). The combined organic phase was dried over anhydrous MgSO_4_, filtered and concentrated in vacuo. The residue was subjected to column chromatography (P.E./EtOAc 9.5:0.5), the solvent was evaporated and the residue was washed with hexane to yield the desired anhydride.

4-Amino-5,7-dimethylisobenzofuran-1,3-dione (**6a**)**:** From aminophthalimide **4** (80 mg). Yellow solid (47 mg, 82%). **^1^H NMR** (200 MHz, DMSO-*d*_6_) δ 7.34 (s, 1H, ArH), 6.38 (bs, 2H, NH_2_), 2.39 (s, 3H, CH_3_), 2.21 (s, 3H, CH_3_). **^13^C NMR** (50 MHz, DMSO-*d*_6_) δ 164.41, 163.52, 145.21, 139.79, 131.94, 126.37, 124.46, 107.69, 17.30, 15.91. **ESI-HRMS** *m*/*z* for C_10_H_8_NO_3_ [M-H]^−^ calcd. 190.0509, found 190.0487.

4-(Hexylamino)isobenzofuran-1,3-dione (**6b**)**:** From aminophthalimide **5a** (91 mg). Yellow solid (13 mg, 17%). **^1^H NMR** (400 MHz, CDCl_3_) δ 7.59 (t, *J* = 7.5 Hz, 1H, H-6), 7.15 (d, *J* = 7.1 Hz, 1H, H-7), 6.94 (d, *J* = 8.5 Hz, 1H, H-5), 6.12 (bs, 1H, NH), 3.29 (t, *J* = 7.1 Hz, 2H, NHC*H*_2_), 1.69 (p, *J* = 7.3 Hz, 2H, NHCH_2_C*H*_2_), 1.47–1.19 (m, 8H, -CH_2_-), 0.90 (t, *J* = 7.0 Hz, 3H, CH_3_). **^13^C NMR** (101 MHz, CDCl_3_) δ 165.26, 163.59, 148.06, 137.87, 132.06, 117.06, 112.77, 109.82, 42.93, 31.59, 29.16, 26.71, 22.68, 14.13. **ESI-HRMS** *m*/*z* for C_15_H_20_NO_4_^−^ [M+MeO]^−^ 278.1398, found 278.1368.

4-(Hexylamino)-5,7-dimethylisobenzofuran-1,3-dione (**6c**)**:** From aminophthalimide **5b** (99 mg). Yellow solid (61 mg, 74%). **^1^H NMR** (200 MHz, CDCl_3_) δ 7.19 (s, 1H, ArH), 5.57 (bs, 1H, NH), 3.46 (t, *J* = 7.0 Hz, 2H, NHC*H*_2_), 2.49 (s, 3H, CH_3_), 2.40 (s, 3H, CH_3_), 1.69–1.50 (m, 2H, NHCH_2_C*H*_2_), 1.48–1.09 (m, 8H -CH_2_-), 0.88 (t, *J* = 6.2 Hz, 3H, CH_2_C*H*_3_). **^13^C NMR** (50 MHz, CDCl_3_) δ 165.10, 163.02, 147.49, 142.05, 133.92, 129.25, 126.20, 112.65, 47.06, 31.60, 31.10, 26.47, 22.66, 20.01, 17.09, 12.85. **ESI-HRMS** *m*/*z* for C_16_H_20_NO_3_ [M-H]^-^ calcd 274.1448, found 274.1440.

## 4. Conclusions

A series of 3-aminophthalates (**1a**–**c**) have been prepared in situ via alkaline hydrolysis of the corresponding 3-aminophthalic acid anhydrides. The latter are the hydrolysis products of the analogous substituted 3-aminophthalimides, which derive from *N*-alkylation and/or Suzuki methylation of the (brominated) parent phthalimide. 3-Aminophthalates’ fluorescence is strongly quenched by ethyltriphenylphosphonium cation, with dynamic rate constants in the order of 10^9^ M^−1^s^−1^, close to the diffusion limit. The substituent effects on the quenching efficiency are consistent with a mechanism involving electron transfer from the electron-rich aminophthalic unit to the electron-deficient phosphonium moiety. Accordingly, the chemiluminescence of luminol is indeed significantly reduced in the presence of phosphonium ions. The possible occurrence of this effect has to be taken into consideration when designing mitotropic drugs or diagnostic agents based on fluorescent or chemiluminescent emitters.

## Figures and Tables

**Table 1:** Quenching rate constants obtained by: ^a^ steady state fluorescence (k_q_), ^b^ time resolved fluorescence (k′_q_).

			pH 8 ETPP		pH 8 TMP	
Phthalate	Φ_F_ pH 10	τ_o_ (ns)	k_q_ (M^−1^s^−1^) ^a^	k′_q_ (M^−1^s^−1^) ^b^	k_q_ (M^−1^s^−1^) ^a^	k′_q_ (M^−1^s^−1^) ^b^
**3AP**	0.30	6.1	3.7 × 10^9^	2.5 × 10^9^	2.7 × 10^8^	9.0 × 10^7^
**1a**	0.34	5.7	5.8 × 10^9^	3.4 × 10^9^	-	-
**1b**	0.07	6.2	6.0 × 10^9^	5.4 × 10^9^	-	-
**1c**	0.08	2.5	9.0 × 10^9^	8.4 × 10^9^	-	-

## Data Availability

The data presented in this study are available in the Materials and Methods section of the article, as well as the Supplementary Materials.

## Supplementary Materials

The following are available online, Figure S1: Absorption spectra for compound **1a**–**c** and **3AP** upon increasing amounts of **ETPP**; Figure S2: Emission of **3AP** and **1a**–**c** at pH 10; Figure S3: Emission and fluorescence decays of **3AP** upon increasing amounts of **TMP**; Figure S4: **3AP** signal intensity versus laser energy; Figure S5: **3AP** decays monitored at 700 nm under N_2_, air, and O_2_; Figure S6: Absorption and emission spectra of **3AP**; Figure S7: Cyclic voltammograms of **3AP** and **ETPP**; Figure S8: ^1^H, ^13^C NMR spectra.

## Sample Availability

Limited samples of all synthesized compounds are available from the authors.
