# Supplementary material for: Modulation by Phosphonium Ions of the Activity of Mitotropic Agents Based on the Chemiluminescence of Luminols
Source: Molecules. 2022 Feb 12;27(4):1245. doi: 10.3390/molecules27041245 (PMC8877776; doi:10.3390/molecules27041245)
Supplement: Supplementary file 1 [file molecules-27-01245-s001.zip › molecules-1583812-supplementary.pdf]

SUPPLEMENTARY MATERIAL

for

Modulation by Phosphonium Ions of the Activity of Mitotropic Agents Based on the Chemiluminescence of Luminols

by

Gemma M. Rodríguez-Muñiz,[1] Theodoros Mikroulis,[2] Anna Pantelia,[2] Georgios Rotas,[2,3] M. Consuelo Cuquerella,[1] Georgios C. Vougioukalakis[2]* and Miguel A. Miranda[1]*

1 Instituto de Tecnología Química UPV-CSIC, Universitat Politècnica de València, Camino de Vera s/n, 46022 València, Spain; gemrodmu@itq.upv.es (G. M. R.-M.); xecual@gmail.com (M. C. C.)

2 Laboratory of Organic Chemistry, Department of Chemistry, National and Kapodistrian University of Athens, Panepistimiopolis, 15771 Athens, Greece; theo.mik0@gmail.com (T. M.); annapantelia@gmail.com (A. P.); rotasgiorgos@hotmail.com (G. R.)

* Correspondence: vougiouk@chem.uoa.gr Tel.: +30 210 7274230. Fax: +30 210 7274761 (G. C. V.) mmiranda@qim.upv.es Tel.: +34 953977807. Fax: +34963877807 (M. A. M.)

Figure S1. Absorption spectra for compound **1a-c** and **3AP** upon increasing amounts of **ETPP**

Figure S2. Emission of **3AP** and **1a-1c** at pH 10 to determine the fluorescence quantum yield and Stern-Volmer plots of the emission spectra and fluorescence decays for compounds **1a-c** and **3AP** obtained in PBS.

Figure S3. Emission and fluorescence decays of **3AP** upon increasing amounts of **TMP**

Figure S4. **3AP** signal intensity *versus* laser energy

Figure S5. **3AP** decays monitored at 700 nm under N_2, air and O_2

Figure S6. Absorption and emission spectra of **3AP**

Figure S7. Cyclic voltammograms of **3AP** and **ETPP**

Figure S8. ^{1}H, ^{13}C NMR spectra.

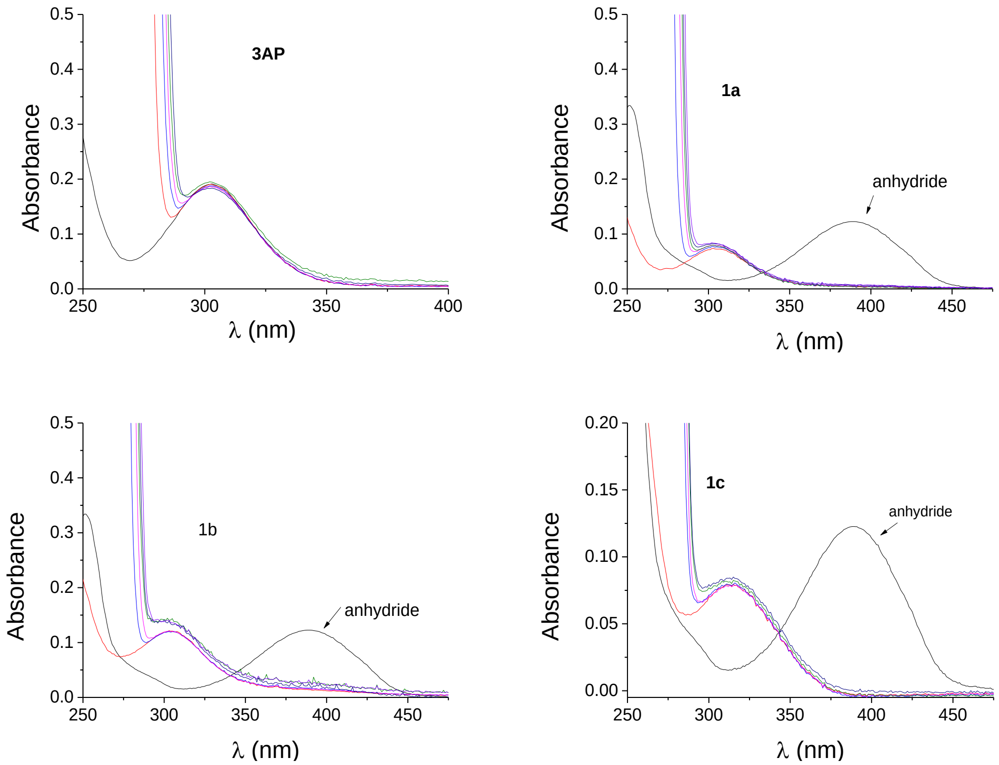

Figure S1. Absorption spectra for compounds **1a-c** and **3AP** obtained in PBS (0.1 mM) in the presence of increasing amounts of **ETPP** (0-42.1 mM. Color codes: 0 mM black line, 4.6 mM red line, 11.4 mM blue line, 22.2 mM pink line, 32.4 mM green line, 42.1 mM navy blue line).

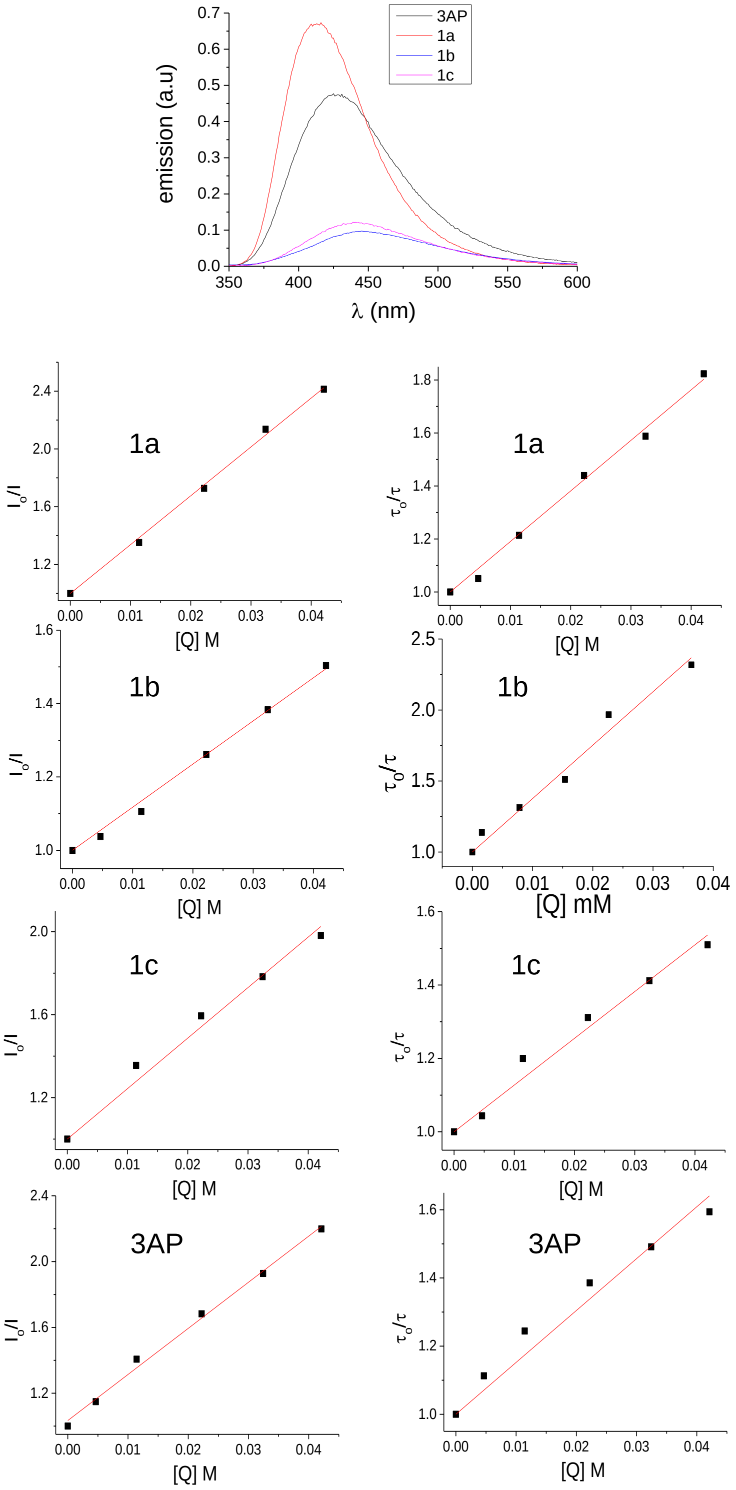

Figure S2. Emission of **3AP** and **1a-1c** at pH 10 to determine the fluorescence quantum yield and Stern-Volmer plots of the emission spectra (left) and fluorescence decays (right) for compounds **1a-c** and **3AP** obtained in PBS (0.1 mM) in the presence of increasing amounts of **ETPP** (0-42.1 mM)

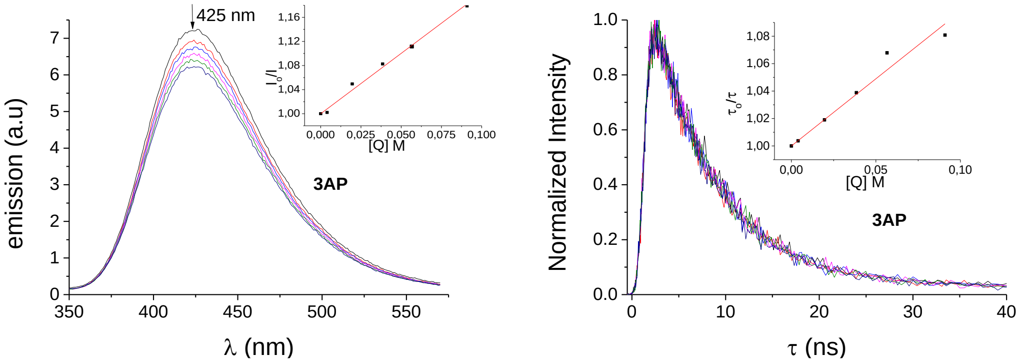

Figure S3. Emission spectra (left) and fluorescence decays (right) for compound **3AP** obtained in PBS (0.1 mM) in the presence of increasing amounts of **TMP** (0-90.9 mM). The corresponding Stern-Volmer plots are shown in the insets.

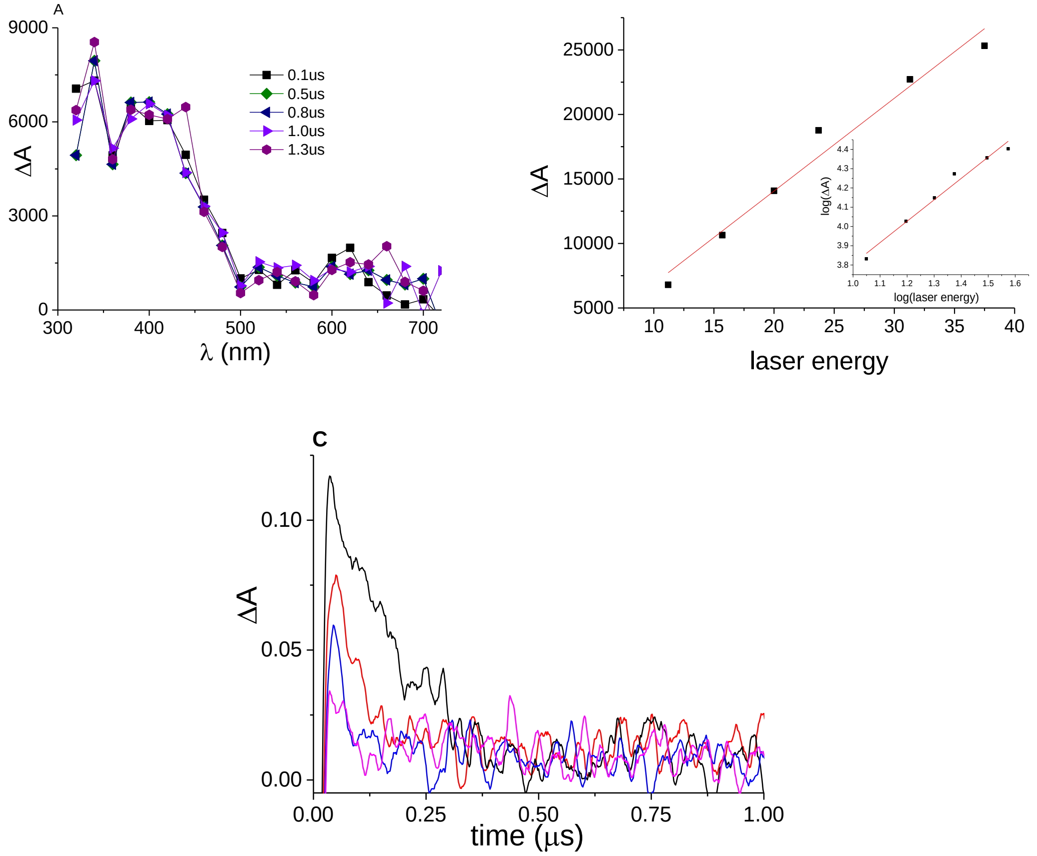

Figure S4. A) Transient absorption spectra of a N_2O-bubbled PBS solution of **3AP** at pH 8 at different times after the 355 nm laser pulse. B) **3AP** signal intensity *versus* laser energy (mJ pulse^{-1}). Inset: Log–log representation. C) Decays monitored at 700 nm under N_2 of the solvated electron quenched by **ETPP** (Color code is: 0 mM black line, 0.24 mM red line, 0.4 mM blue line, 0.8 mM pink line)

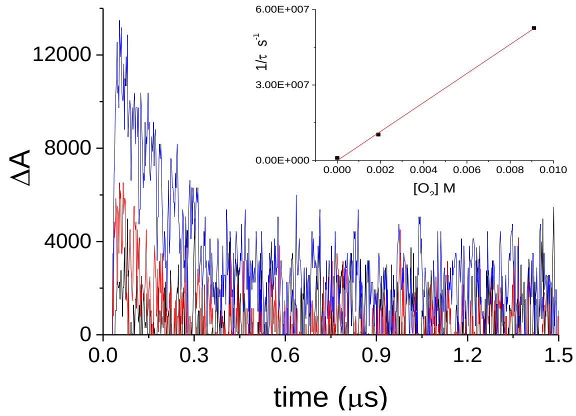

Figure S5. Quenching of ^{3}**AP*** by oxygen as revealed by the decays monitored at 700 nm under N_2 (blue), air (red), or O_2 (black). Inset: Stern-Volmer plot.

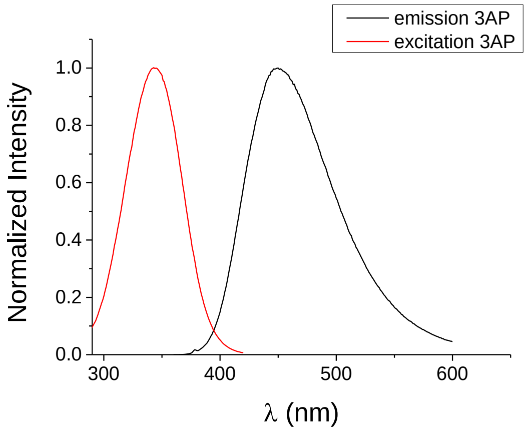

Figure S6. Absorption and emission spectra of **3AP** in phosphate buffer pH 8.

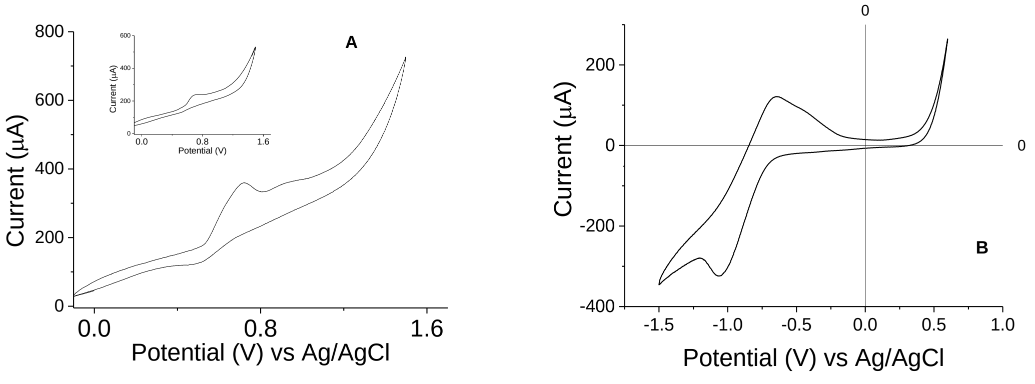

Figure S7. Cyclic voltammograms of A) **3AP** (1 mM, in PBS pH 8, using 0.1 M $LiClO_4$ as electrolyte), inset cyclic voltammogram of PBS pH 8/ 0.1 M $LiClO_4$ as electrolyte and B) **ETPP** (1 mM in DMF, using Bu_4NI as electrolyte). Scan rate: 0.05 Vs^{-1}

Figure S8. ^{1}H, ^{13}C NMR spectra.

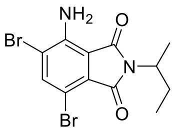

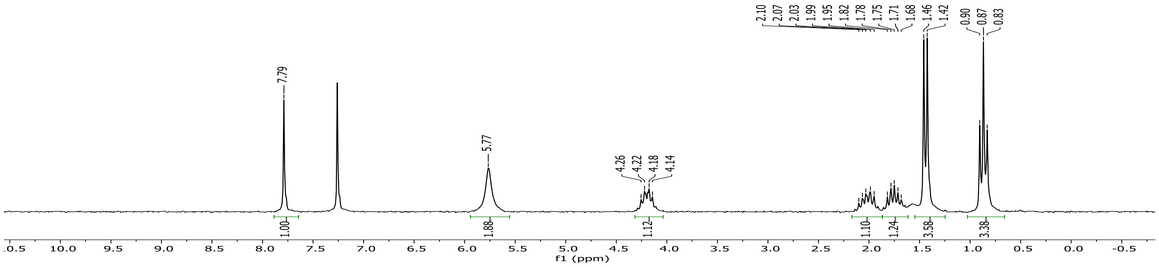

Figure S8a: ^{1}H-NMR (200 MHz, $CDCl_3$) spectrum of **3**.

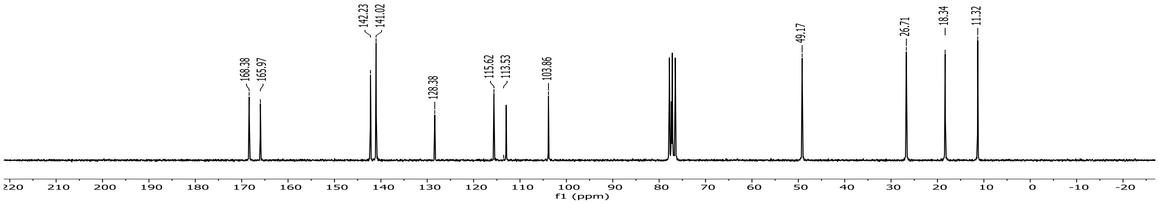

Figure S8b: ^{13}C-NMR (50 MHz, $CDCl_3$) spectrum of **3**.

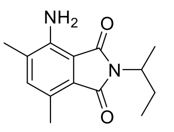

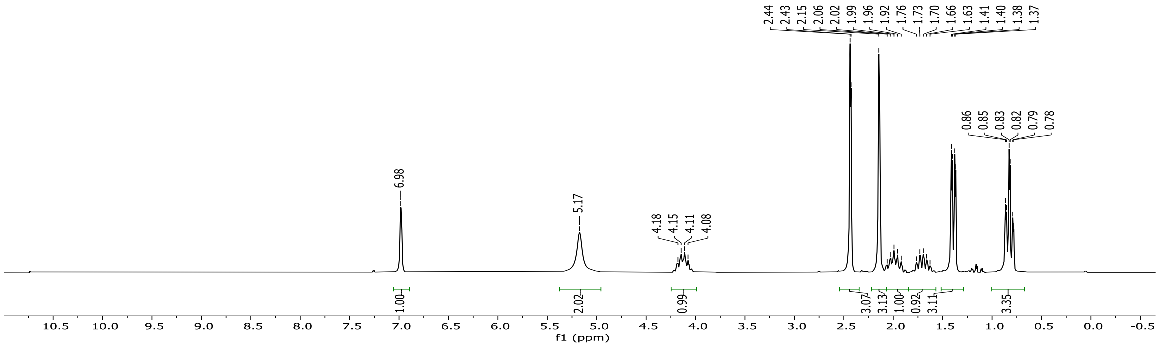

Figure S8c: ^{1}H-NMR (200 MHz, $CDCl_3$) spectrum of **4**.

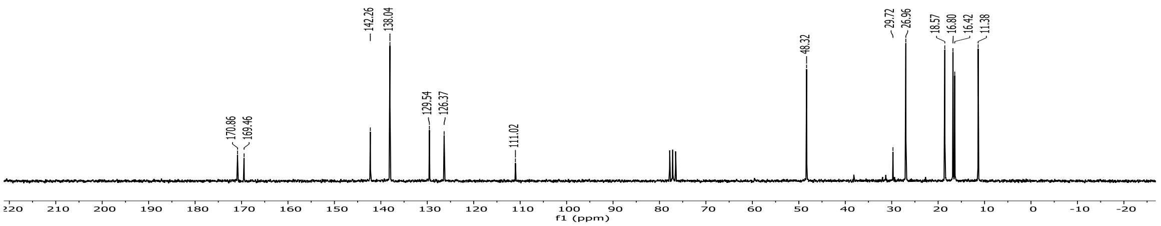

Figure S8d: ^{13}C-NMR (50 MHz, $CDCl_3$) spectrum of **4**.

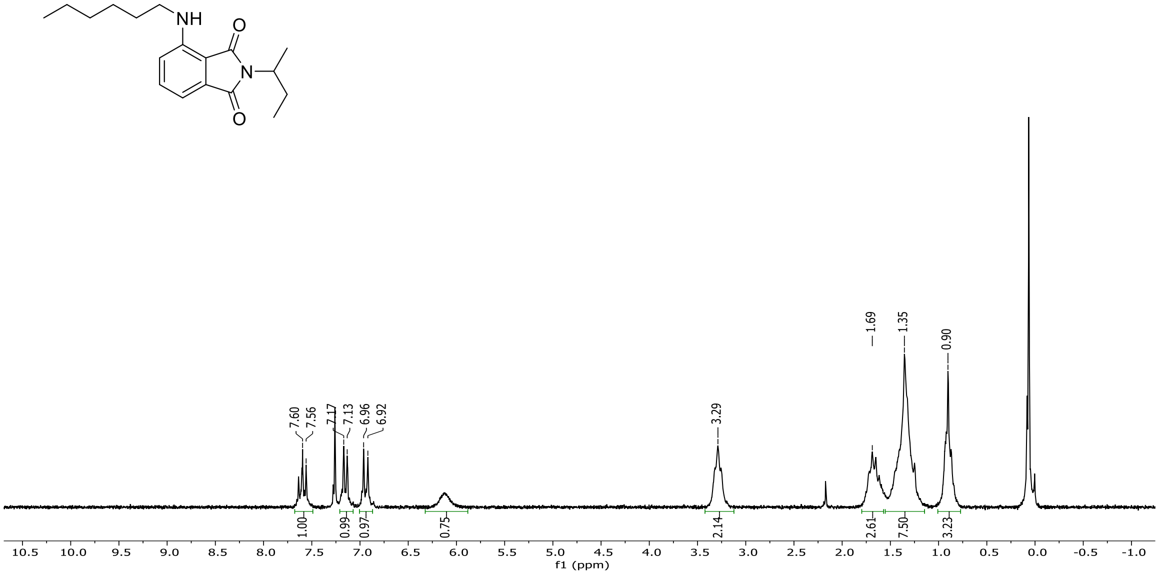

Figure S8e: ^{1}H-NMR (200 MHz, $CDCl_3$) spectrum of **5a**.

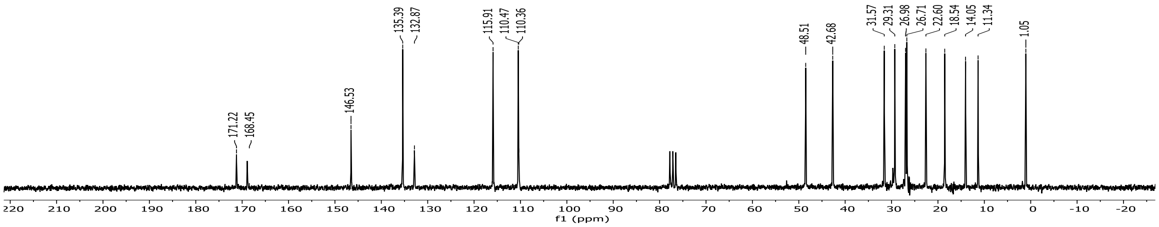

Figure S8f: ^{13}C-NMR (50 MHz, $CDCl_3$) spectrum of **5a**.

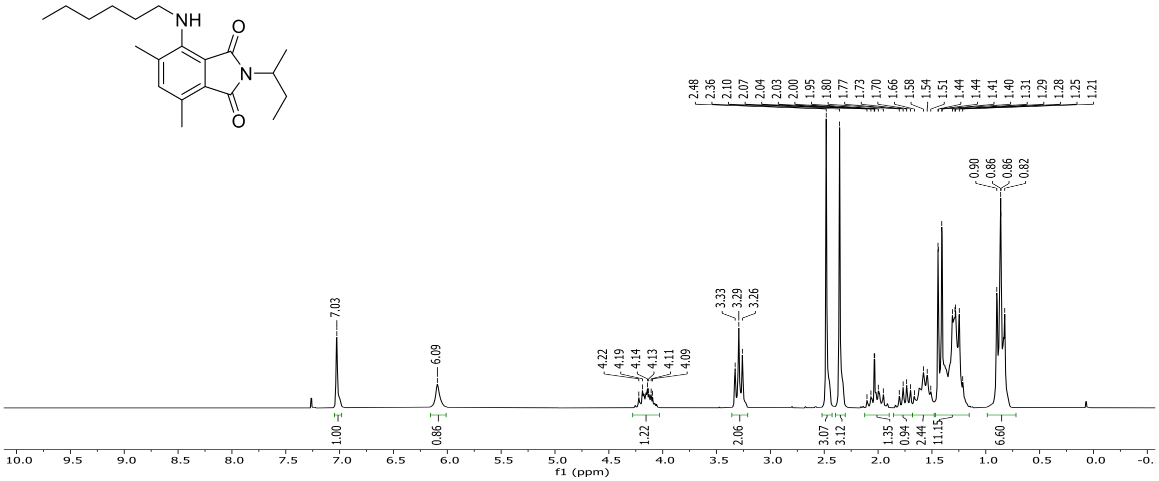

Figure S8g: ^{1}H-NMR (200 MHz, $CDCl_3$) spectrum of **5b**.

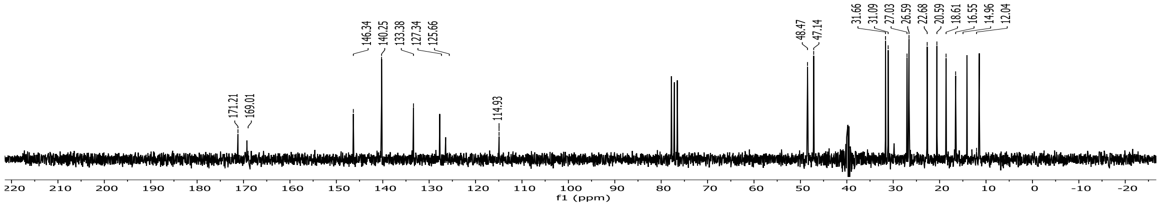

Figure S8h: ^{13}C-NMR (50 MHz, $CDCl_3$) spectrum of **5b**.

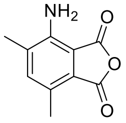

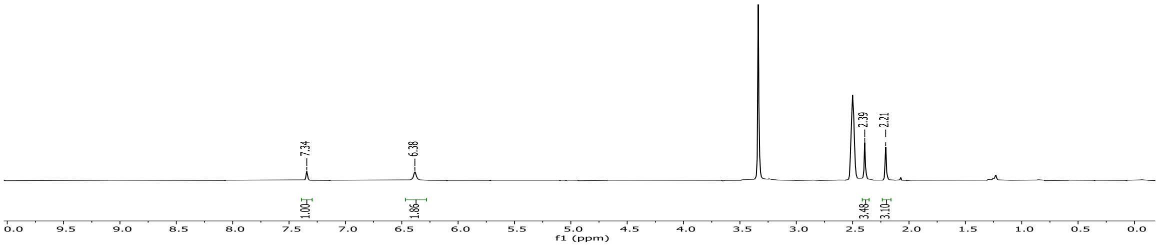

Figure S8i: ^{1}H-NMR (200 MHz, DMSO-d$_6$) spectrum of **6a**.

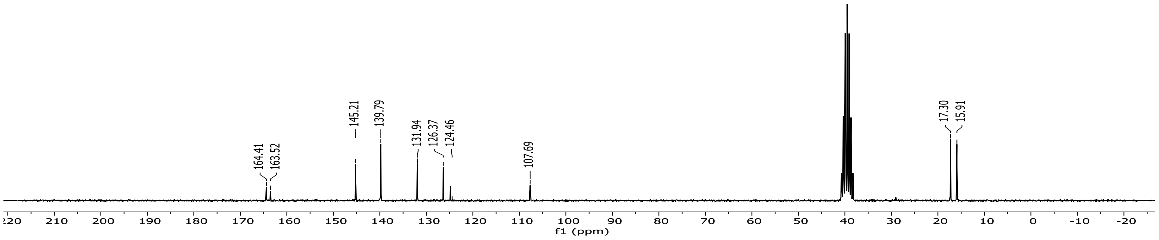

Figure S8j: ^{13}C-NMR (50 MHz, DMSO-d$_6$) spectrum of **6a**.

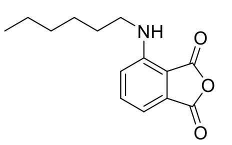

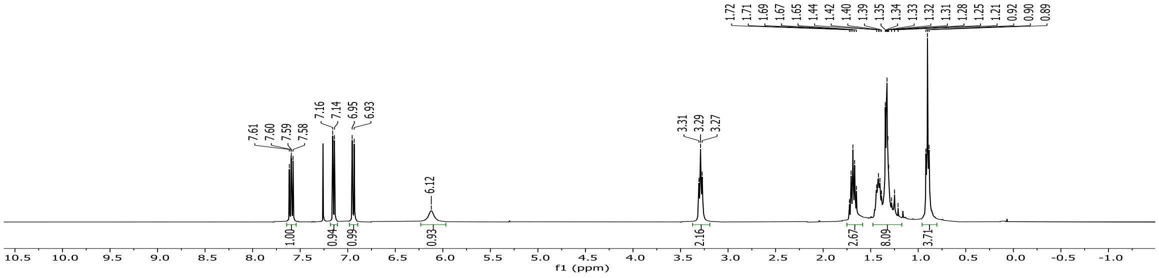

Figure S8k: ^{1}H-NMR (400 MHz, $CDCl_3$) spectrum of **6b**.

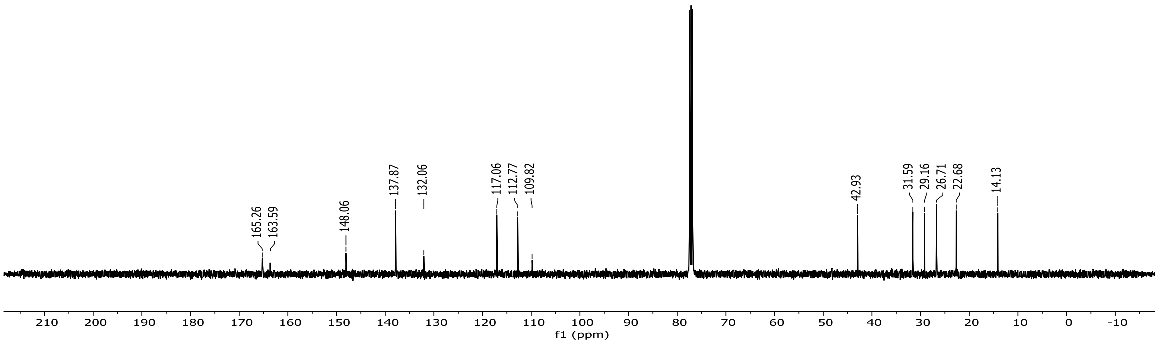

Figure S8l: ^{13}C-NMR (101 MHz, $CDCl_3$) spectrum of **6b**.

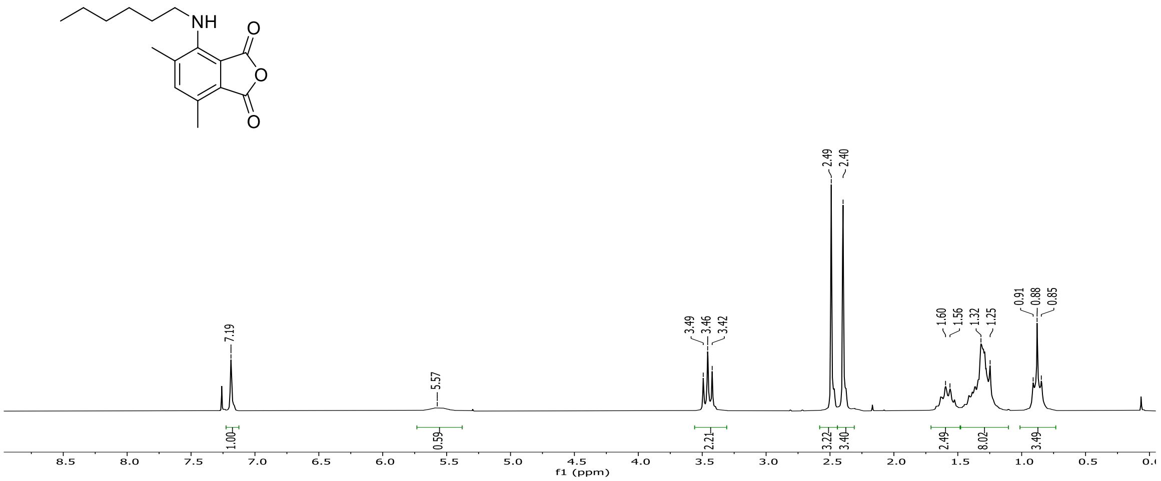

Figure S8m: ^{1}H-NMR (200 MHz, $CDCl_3$) spectrum of **6c**.

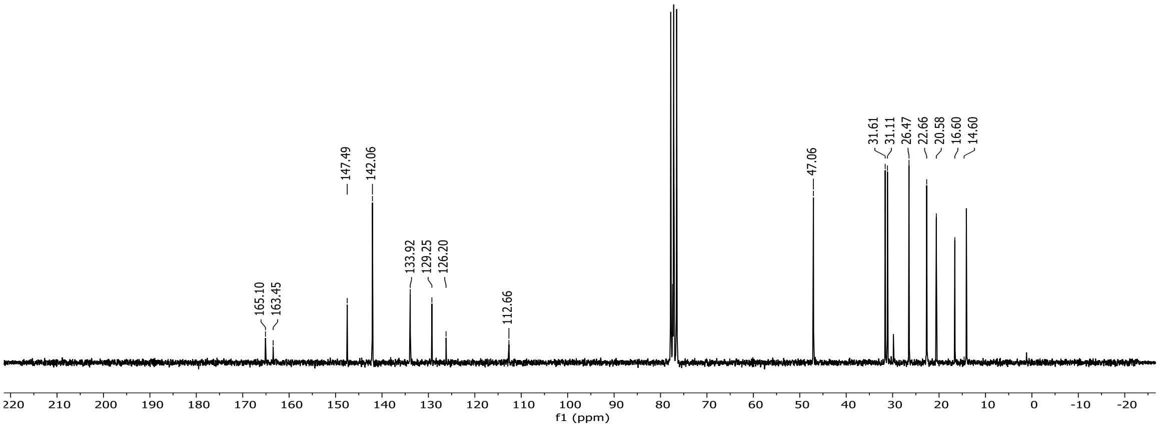

Figure S8n: ^{13}C-NMR (50 MHz, $CDCl_3$) spectrum of **6c**.
